# Anxiety and depression in first-year university students: the role of family and social support

**DOI:** 10.3389/fpsyg.2024.1462948

**Published:** 2024-11-22

**Authors:** Andrea Estrella-Proaño, María F. Rivadeneira, Jéssica Alvarado, Matías Murtagh, Susana Guijarro, Lidia Alomoto, Glenda Cañarejo

**Affiliations:** ^1^Faculty of Nursing, Department of Nutrition and Dietetics, Pontificia Universidad Católica del Ecuador, Quito, Ecuador; ^2^Institute of Public Health, Faculty of Medicine, Pontificia Universidad Católica del Ecuador, Quito, Ecuador; ^3^Postgraduate in Family and Community Medicine, Faculty of Medicine, Pontificia Universidad Católica del Ecuador, Quito, Ecuador; ^4^Faculty of Psychology, Pontificia Universidad Católica del Ecuador, Quito, Ecuador

**Keywords:** anxiety, depression, family dysfunction, social support, university students

## Abstract

**Background:**

Adolescents and young are one of the population groups with the highest prevalence of anxiety and depression worldwide. Few studies address this problem in young university students. This study aimed to analyze the prevalence of anxiety and depression in first-year university students and its association with family functionality and social support.

**Methods:**

A cross-sectional study was carried out on 847 students from five cities in Ecuador, between 18 and 25 years of age, who were beginning their university career. Anxiety and depression symptoms were measured with the Hospital Anxiety and Depression Scale, family functionality with the FF-SIL Test, and social support with the Medical Outcomes Study Scale. The sociodemographic characteristics of the participants were also measured. Bivariate and multivariate data analyzes were performed using logistic regression.

**Results:**

The 19.7% of the students presented anxiety, while 24.7% presented depression. Female students, students with poor economic status, and married/in union students had a higher prevalence of anxiety and depression. Family dysfunction and lack of global social support were significantly associated with a higher prevalence of anxiety (OR 1.93 95% CI 1.20–3.10; OR 1.99 95% CI 1.19–3.33, respectively) and depression (OR 1.87 95% CI 1.16–3.01; OR 2.2 95% CI 1.35–2.57, respectively), regardless of the student’s economic situation.

**Conclusion:**

Social support and family functionality play an important role in the prevention of anxiety and depression in first-year university students. It is necessary to establish mental health policies and strategies in this underserved population group that strengthen social support and family functionality.

## 1 Introduction

Anxiety and depression are two mental health problems with high prevalence and impact among adolescents and young adults (Van der Walt et al., 2020; Ibrahim et al., 2013). The World Health Organization states that depression is one of the leading causes of disability and a significant contributor to the global burden of disease (World Health Organization, 2018). Studies suggest that the prevalence of depression could reach from 10 to 85% in the university population, with a mean of 30.6% (Ibrahim et al., 2013). Likewise, anxiety could get a prevalence of 20 to 50% in university students (Ramón-Arbués et al., 2020; Bassols et al., 2014; Haldorsen et al., 2014), with higher percentages in the first years of university life (Bassols et al., 2014). Students who begin university studies face stressful situations, excessive demands from their environment, psychological exhaustion, and dissatisfaction, which, in addition to individual, family, psychosocial and academic life variables, lead to a deterioration of their mental health (Abdallah and Gabr, 2014; Beiter et al., 2015; Islam et al., 2020). Anxiety and depression are multifactorial. Among the many factors that affect students’ mental health, previous mental disorders stand out (Amaro et al., 2024), including underdiagnosed ones, as well as adverse factors in childhood (Mall et al., 2018), or current stressors (such as abuse, illness, death of a family member, etc.). Sleep disturbances have also been associated to depression and anxiety in students (Baldini et al., 2024; Roldán-Espínola et al., 2024). On the other hand, the weakening of social relationships, the increase in exposure to different forms of violence (Chen et al., 2024), as well as greater demands in terms of competitiveness and productivity in university environments could lead to greater mental distress (Amaro et al., 2024). Other predictors of mental health deterioration would include those related to body image, self-esteem, pressure to succeed and relationships with friends and family (Emmerton et al., 2024).

Start university education includes some modifications in students’ lives, including increased independence and responsibility, family and friend separation, changes in adult supervision, academic demands, and new social networks. Students perceive these changes as stressful events that require adaptation. They have to adjust to a novel educational and social environment and fulfill their family expectations and their projections about academic life (Islam et al., 2020). One of the critical points in the process of student adaptation to their new reality is the family. Family functionality is a group of qualities that allow its members to function as a system, transforming in the face of change, which favors flexibility of adaptation in the face of crises and contributes to the development of its members (Epstein et al., 1983). There are six dimensions of family functionality: problem-solving capacity, communication, roles, affective response, affective involvement, and behavioral control (Epstein et al., 1983). A dysfunctional family, which presents distortions in contact or any other dimensions, generates a hostile environment among the system members, which minimizes its primary role of protection and guidance. Family dysfunctionality is associated with health problems, including anxiety and depression (Shao et al., 2020; Wang et al., 2020).

Depression and anxiety in young adults are also related to a lack of social support. Many students feel little backing from their either family or friends during their university studies. Several theories claim that good social support is a protective factor against the adverse effects of stress (Wang et al., 2020; Cohen and Wills, 1985; Cohen, 1992). Studies mention that the lack of social support associates with the appearance of somatic symptoms, anxiety, insomnia, social dysfunction, and severe depression, in university students (Alsubaie et al., 2019; Wang et al., 2014), on the other hand, social support would be a protective element against suicidal ideation in adolescents (Mackin et al., 2017). Previous studies point to a significant association between the role of family and society, with the presence of mental health problems, such as anxiety and depression in adolescents and young adults (Islam et al., 2020; Epstein et al., 1983; Shao et al., 2020). However, studies in the population recently entering university life are scarce, especially in low to middle-income contexts, as is the case of Ecuador, which also has a Hispanic population culturally characterized by a closer relationship with their nuclear families. Based on this conceptual framework, we conducted the present study to analyze the association between symptoms of anxiety and depression with family functionality and social support in first-year university students. We hope that this evidence contributes to understanding the role of family and social support in this population group.

## 2 Materials and methods

### 2.1 Study design and population

We conducted an analytical cross-sectional study on first-year undergraduate university students. The population of this study was 2,693 newly admitted students from a private university in Ecuador, with campuses in five cities in the country: Quito, Ibarra, Santo Domingo, Portoviejo and Chone. This study was carried out in 2019. A representative sample of 847 university students was calculated, for an expected prevalence of 30.6% of depression in university students, according to Ibrahim et al. (2013), for a confidence level 95 and 2% sampling error.

We select the students by quota sampling from a university enrollment list to represent the number of students in each city. Students were randomly selected in each of the careers offered 2019. We recruited the students in their classrooms with the prior authorization of the teachers and faculty authorities. Those who agreed to participate signed the informed consent form and answered the survey. Inclusion criteria were: age 18 and 25 years, first-time students, and accepting to participate voluntarily. We excluded students with visual, hearing and physical disabilities as well as students diagnosed with chronic non-communicable diseases (hypertension, diabetes) and mental health diseases (attention deficit disorders, schizophrenia, etc.) to reduce confounding biases.

### 2.2 Variables and measures

We measured anxiety and depression symptoms by applying the Hospital Anxiety and Depression Scale (HADS) (Zigmond and Snaith, 1983), an instrument for self-assessment and screening of anxiety and depression; it allows the exclusion of somatic symptoms such as insomnia, fatigue, and loss of appetite, which could bias the investigation. It comprises two subscales of seven items each: HAD A for anxiety and HAD D for depression, with a score from zero to 21, to define anxiety and depression. We considered the following cut-off points: 0–7 no case, 8–10 doubtful cases, and 11 or more probable cases (Zigmond and Snaith, 1983). Questionable and potential instances of anxiety and depression were categorized as symptomatic of anxiety or depression, while non-cases were renamed as no anxiety or depression. This instrument has also been used to measure anxiety and depression in adolescent and young populations (Kathem et al., 2021). For this study, the HADS questionnaire reached an adequate level of reliability (Cronbach’s alpha 0.89).

Family functioning was measured using the FF-SIL Test, validated by Ortega et al. (1999). This instrument has demonstrated adequate reliability and internal consistency. It is easy to apply and understand for any level of schooling. It consists of seven dimensions that assess the following: cohesion, harmony, communication, affectivity, roles, permeability, and adaptability; it measured each size through two questions, with a Likert scale of five responses (seldom, rarely, sometimes, many times, usually). We classified the families according to the score obtained as functional (70 to 57 points), or moderately functional (56 to 43 points), dysfunctional (42 to 28 points), and severely dysfunctional (56 to 14 points) (Ortega et al., 1999). The FF-SIL test obtained for this study, a Cronbach’s alpha of 0.91, reaching optimal levels of reliability.

We used The Medical Outcomes Study (MOS) designed by Sherbourne and Stewart (1991), to measure social support. This questionnaire has good psychometric properties and can be applied at the community level. It assesses four dimensions of social support: (a) affective (demonstration of love, affection, and empathy), (b) positive social interaction (possibility of having people to communicate with), (c) instrumental (option of domestic help), and (d) emotional (opportunity of advice, counsel, and care). It consists of 20 items, scoring from one (never) to five (always). In this study, it was classified according to the score obtained in each dimension into has social support or has lack of social support (lack of support when the score was less than 19 on the global index, three on affective support, four on instrumental support, and eight on emotional support) (Sherbourne and Stewart, 1991). The reliability test (Cronbach’s alpha) for the MOS questionnaire applied in this study was 0.84, showing good internal consistency (Moser et al., 2012).

In addition, we gather the following sociodemographic information: sex (male/female), age (adolescents: 18–19 years, young adults: 20–25 years), marital status (single, married, common-law, widowed), economic status according to students’ perception (regular, good, very good, bad, deplorable), living situation (parents, single parent, other relatives, single, partner) and migratory status (yes/no) through a self-filled survey.

### 2.3 Statistical analysis

We performed a descriptive analysis for all variables. We conducted a bivariate analysis by logistic regression to associate anxiety and depression symptoms with family functionality and social support. Odds ratios with 95% confidence intervals were obtained. We also made a multivariate analysis between anxiety and depression with family functionality and social support, adjusted for sex, age, marital status, living with, and economic situation. We used the SPSS version 25 statistical package to perform the analysis data.

### 2.4 Ethics approval

The Human Research Ethics Committee from the Pontificia Universidad Católica del Ecuador approved the study. We obtained an informed consent letter from all participants.

## 3 Results

Table 1 shows the sociodemographic characteristics of the included students. The 24.7% (*n* = 209) of the students exhibit a probable case of depression, 12.4% (*n* = 105) have a doubtful case and 62.9% (*n* = 533) have no depression. About depression, females present 1.41 times more prevalence (95% CI 1.06–1.88; *p* < 0.05) compared to men, young adults have 34% less prevalence than adolescents (OR 0.66; 95% CI 0.50–0.89; *p* < 0.01), and married or cohabiting students have a 6.60 times higher prevalence of this disease (95% CI 2.64–16.47; *p* < 0.01). Students in poor economic situations were 46% more likely to present anxiety symptoms compared to students in good/very good economic conditions (OR 1.45, 95% CI 1.10–1.95; *p* < 0.01) (Table 2).

**Table 1:** Sociodemographic characteristics of first-year students (*n* = 847).

Characteristics	Frequency	Percentage
Sex		
Male	358	42.3
Female	489	57.7
Age		
Adolescents (18–19 years)	496	58.6
Young adults (20–25 years)	351	41.4
Marital status		
Single	818	96.6
Married	21	2.5
Cohabiting	7	0.8
Widowed	1	0.1
Economic situation (perceived)		
Poor	445	52.5
Good	360	42.5
Very good	20	2.4
Bad	20	2.4
Deplorable	2	0.2
Living situation		
Parents	440	51.9
Mother	199	23.5
Relatives	72	8.5
Alone	53	6.3
Mother and stepfather	27	3.2
Father	25	3.0
Roommate	21	2.5
Partner	8	0.9
Father and stepmother	2	0.2
Migrate to study		
No	654	77.2
Yes	193	22.8

**Table 2:** Association between anxiety and depression with sociodemographic characteristics of students (*n* = 847).

	Anxiety		Depression			
	Yes (%)	No (%)	OR (IC 95%)	Yes (%)	No (%)	OR (IC 95%)
Sex						
Male	153 (42.7)	205 (57.3)	Reference	116 (32.4)	242 (67.6)	Reference
Female	256 (52.4)	233 (47.6)	1.47 (1.11–1.93)^**^	198 (40.5)	291 (59.5)	1.41 (1.06–1.88)*
Age						
Adolescents	235 (47.4)	261 (52.6)	Reference	203 (40.9)	293 (59.1)	Reference
Young adults	174 (49.6)	177 (50.4)	1.09 (0.83–1.43)	111 (31.6)	240 (68.4)	0.66 (0.50–0.89)**
Marital status						
Single	387 (47.3)	431 (52.7)	Reference	292 (35.7)	526 (64.3)	Reference
Married/cohabiting	22 (78.6)	6 (21.4)	4.08 (1.63–10.17)^**^	22 (78.6)	6 (21.4)	6.60 (2.64–16.47)**
Economic situation						
Very good/Good	166 (43.7)	214 (56.3)	Reference	122 (32.1)	258 (67.9)	Reference
Poor	227 (51.0)	218 (49.0)	1.34 (1.02–1.76)*	182 (40.9)	263 (59.1)	1.46 (1.10–1.95)**
Bad/deplorable	16 (72.7)	6 (27.3)	3.43 (1.32–8.98)*	10 (45.5)	12 (54.5)	1.76 (0.74–4.19)
Living situation						
Parents	203 (46.1)	237 (53.9)	Reference	164 (37.3)	276 (62.7)	Reference
Mother/father	100 (44.6)	124 (55.4)	0.94 (0.68–1.30)	74 (33.0)	150 (67.0)	0.83 (0.59–1.17)
Mother/Father and stepfather/stepmother	15 (51.7)	14 (48.3)	1.25 (0.59–2.65)	15 (51.7)	14 (48.3)	1.80 (0.85–3.83)
Relatives	41 (56.9)	31 (43.1)	1.54 (0.93–2.55)	29 (40.3)	43 (59.7)	1.14 (0.68–1.89)
Partner/roommate	19 (65.5)	10 (34.5)	2.21 (1.01–4.88)*	15 (51.7)	14 (48.3)	1.80 (0.85–3.83)
Alone	31 (58.5)	22 (41.5)	1.65 (0.92–2.93)	17 (32.1)	36 (67.9)	0.80 (0.43–1.46)
Migrate to study						
No	308 (47.1)	346 (52.9)	Reference	243 (37.2)	411 (62.8)	Reference
Yes	101 (52.3)	92 (47.7)	1.23 (0.89–1.70)	71 (36.8)	122 (63.2)	0.98 (0.71–1.37)

^*^p-value statistically significant *p* < 0.05.

^**^p-value statistically significant *p* < 0.01.

The 46.9% (*n* = 397) of university students belong to a functional family, 39.8% (*n* = 337) to a moderately functional family, 11% (*n* = 93) to dysfunctional family, and 2.4 (*n* = 20) to severely dysfunctional family. Students with dysfunctional families have a 2.03 times higher prevalence of anxiety (95% CI 1.28–3.24) and 2.07 times higher prevalence of depression (95% CI 1.31–3.26) compared to students with functional families (*p* < 0.01) (Table 3). The association between anxiety and family dysfunctionality remains significant (OR 1.93, 95% CI 1.20–3.10; *p* < 0.01), independently of other variables. The association between depression and family dysfunction remains significant after adjusting sociodemographic variables (OR 1.87, 95% CI 1.16–3.01, *p* < 0.05).

**Table 3:** Association between anxiety and depression with family functionality in first-year university students.

	Anxiety				Depression			
	Yes (%)	No (%)	OR (IC 95%)	Adjusted OR^a^ (IC 95%)	Yes (%)	No (%)	OR (IC 95%)	Adjusted OR^a^ (IC 95%)
Functional	178 (44.8)	219 (55.2)	Reference	1.07 (0.80–1.45)	135 (34.0)	262 (66.0)	Reference	Reference
Moderately functional	162 (48.1)	175 (51.9)	1.13 (0.85–1.52)	1.93 (1.20–3.10)**	121 (35.9)	216 (64.1)	1.08 (0.80–1.47)	1.06 (0.77–1.45)
Dysfunctional	58 (62.4)	35 (37.6)	2.03 (1.28–3.24)**	1.32 (0.52 – 3.35)**	48 (51.6)	45 (48.4)	2.07 (1.31–3.26)**	1.87 (1.16 – 3.01)**
Severely dysfunctional	11 (55.0)	9 (45.0)	1.50 (0.61–3.70)	1.07 (0.80–1.45)	10 (50.0)	10 (50.0)	1.94 (0.78–4.77)	1.77 (0.70–4.46)

^Bivariate and multivariate logistic regression (*n* = 847).^

^**^p-value statistically significant *p* < 0.01.

^aOR adjusted for sex, age, marital status, and economic and living situation.^

The 88.35% of the students (*n* = 721) report having social support. Table 4 shows the association between social support dimensions with anxiety and depression. Low social support increases anxiety symptoms 2.64 times (95% CI 1.66–4.18; *p* < 0.01), deficient emotional support and poor affective support raise anxiety symptoms in 2.25 and 2.24 (95% CI 1.53–3.23; 1.33–3.77; *p* < 0.01), respectively. Lack of instrumental support showed a significant association with anxiety in the bivariate analysis (OR 1.52 95% CI 1.9–2.59; *p* < 0.05), but this association did not remain significant after performing the adjusted analysis (OR 1.50 95% CI 0.83–2.72; *p* < 0.13). Low social interaction is associated with a 2.57 times higher prevalence of anxiety symptoms (95% CI 1.63–4.06; *p* < 0.01). The multivariate analysis exhibit that emotional support, adequate support, and social interaction are positively associated with higher anxiety symptoms. Besides, lack of global social support is remarkably associated with a higher prevalence of anxiety (OR 1.99 95%CI 1.19–3.33; *p* < 0.01) (Table 4).

**Table 4:** Association between anxiety and depression with social support in first-year university students.

	Anxiety				Depression			
	Yes (%)	No (%)	OR (IC 95%)	Adjusted OR^a^ (IC 95%)	Yes (%)	No (%)	OR (IC 95%)	Adjusted OR^a^ (IC 95%)
Global social support								
Yes	334 (46.3)	387 (53.7)	Reference	Reference	255 (35.4)	466 (64.6)	Reference	Reference
Lack of support	66 (69.5)	29 (30.5)	2.64 (1.66–4.18)**	1.99 (1.19–3.33)**	54 (56.8)	41 (43.2)	2.41 (1.56–3.71)**	2.20 (1.36–2.57)**
Emotional support								
Yes	303 (44.7)	375 (55.3)	Reference	Reference	233 (34.4)	445 (65.6)	Reference	Reference
Lack of support	102 (64.6)	56 (35.4)	2.25 (1.57–3.23)**	2.09 (1.44–3.04)**	78 (49.4)	80 (50.6)	1.86 (1.31–2.64)**	1.84 (1.28–2.66)**
Instrumental support								
Yes	370 (47.4)	411 (52.6)	Reference	Reference	285 (36.5)	496 (63.5)	Reference	Reference
Lack of support	36 (62.1)	22 (37.9)	1.82 (0.05–3.15)	1.37 (0.75–2.48)	27 (46.6)	31 (53.4)	1.52 (1.9–2.59)**	1.50 (0.83–2.72)
Social interaction								
Yes	340 (45.7)	404 (54.3)	Reference	Reference	256 (34.4)	488 (65.6)	Reference	Reference
Scarce	65 (68.4)	30 (31.6)	2.57 (1.63–4.06)**	2.40 (1.49–3.85)**	55 (57.9)	40 (42.1)	2.62 (1.70–4.05)**	2.69 (1.71–4.24)**
Emotional support								
Yes	358 (47.2)	401 (52.8)	Reference	Reference	274 (36.1)	485 (63.9)	Reference	Reference
Scarce	46 (66.7)	23 (33.3)	2.24 (1.33–3.77)**	2.04 (1.18–3.51)*	37 (53.6)	32 (46.4)	2.05 (1.25–3.36)**	2.05 (1.22–3.45)**

Bivariate and multivariate logistic regression (*n* = 839).

^*^p-value statistically significant *p* < 0.05.

**p-value statistically significant *p* < 0.01.

^aOR adjusted for sex, age, marital status, and economic and living situation.^

Students with poor global social support have a 2.41 times higher prevalence of depressive symptoms (95% CI 1.56–3.71; *p* < 0.01); meanwhile, lack of emotional support and poor emotional support increase 1.86 and 2.05 times, respectively, the probability of present depressive symptoms (95% CI 1.31–2.64, 1.25–3.3; *p* < 0.01). Poor social interaction was significantly associated with depression. The probability of depression in students with poor social interaction was 2.62 times higher when compared to students who have social interaction (OR 2.62 95% CI 1.70–4.05; *p* < 0.01). Social interaction remained associated with depression after adjustment for sex, age, marital status, economic status, and living situation. Global social support lack was significantly associated with a higher prevalence of depressive symptoms in the multivariate analysis (OR 2.2 95%CI 1.35–2.57, *p* < 0.01) (Table 4).

## 4 Discussion

Mental health currently represents a significant challenge for health systems worldwide due to the increasing incidence of suicide deaths in adolescents and young adults, the latter being the most susceptible to high levels of anxiety and depression (Bassols et al., 2014; Islam et al., 2020). Anxiety and depression are the leading mental health cause of disability and disease burden (World Health Organization, 2018) and are predictors of life level satisfaction (Serin et al., 2020). Our study determined the prevalence of anxiety and depression in students starting university life and its association with family functionality and social support. We found that 24.7% of first-year university students suffer from depression, and 19.7% have anxiety. Worldwide the prevalence of these disorders varies between 10 and 80% for depression and between 20 and 50% for anxiety (Bassols et al., 2014; Ramón-Arbués et al., 2020). A study conducted in Bangladesh found that first-year students have a high prevalence of these mental problems, with 69.5% of depression and 61% anxiety (Islam et al., 2020), explaining the importance of visualizing these problems in students entering university life. Anxiety and depression conduct to negative consequences for university students such as dropout, deplorable academic performance, poor interpersonal relationships, substance abuse, suicidal ideation, and suicide (Serin et al., 2020; Chu et al., 2021). Therefore, early identification of individuals at risk and timely referral are essential for early detection of mental issues in this population.

We report that the prevalence of depression and anxiety is remarkably higher in females, married or cohabiting students, and subjects with a bad/deplorable economic situation. Previous studies ratified the association between depression and anxiety with socioeconomic characteristics. In China, university students with higher financial debts presented higher prevalence of anxiety and depression; lower family income associates with higher anxiety levels (Islam et al., 2020; Rabby et al., 2023). These findings suggest a possible association between socioeconomic inequalities with anxiety and depression in university students. A previous study suggests that, on its own, income inequality could have a role as a “contextual stressor” (Jiang and Probst, 2017) in addition to being perceived as threatening, and therefore, generating greater anxiety and depression (Pickett and Wilkinson, 2007). Likewise, other studies have observed that the greater the economic inequality, the greater the probability of anxiety (King et al., 2024). In the case of university students, perceiving themselves to be in a situation of poverty can also have a social impact: when they considering themselves in an inferior social position, they fear being left behind by their peers, which generates greater anxiety and depression (Kraus et al., 2013; Liu et al., 2024). This sense of relative deprivation makes students more sensitive to interpersonal comparisons and encourages greater competitiveness (King et al., 2022).

Regarding gender differences, other authors have also described a high prevalence of anxiety and depression in females (Faravelli et al., 2013; Leach et al., 2008; Rabby et al., 2023). There are several hypotheses that attempt to explain the higher prevalence of anxiety and depression in women. The results of this study lead us to think about the possibility of gender inequality related to anxiety and depression. Generally, women are the ones who face a greater burden of care in their family, which could be related to the higher prevalence of anxiety and depression, when compared to men; this is particularly important for married or cohabiting women. Female students are also the ones who report lower values of physical and emotional well-being compared to men (Cela-Bertran et al., 2024). On the other hand, women have greater exposure to interpersonal stressors than men (e.g., greater exposure to abuse and violence, and peer judgment) which could be increase the probability of anxiety and depression in women (Anderson et al., 2024). In any case, this finding should be further investigated with other studies that identify the characteristics of women with anxiety and depression.

On the other hand, married or cohabiting students have greater financial burdens, which is an additional stressor to interpersonal, academic and environmental factors. The higher prevalence of anxiety and depression in married or cohabiting students found in this study compared to single students may apply particularly to women. Married young women have been found to have higher levels of anxiety and depression than unmarried women (Dhara et al., 2024). Other studies mention the importance of the quality of marriage for mental health, as turbulent marriages can lead to depression (Bourassa et al., 2015), with greater detriment to the mental health of women compared to men (Kiecolt-Glaser and Newton, 2001).

Another finding of our research is the association between family dysfunction with anxiety and depression; this association remained significant after adjustment for other variables, including economic status. Studies in adolescents and young adults reported that higher family cohesion lowers the risk of developing depressive symptoms and suicidal ideation (Leach et al., 2008); meanwhile, students who are part of dysfunctional families have higher prevalence of depression and anxiety (Bögels and Brechman-Toussaint, 2006; Wang et al., 2020). A positive family environment supports the healthy development of its members; conversely, dysfunctional families often have difficulties in the ability to communicate with each other and solve problems (Miller et al., 2000). Recently admitted students experience some changes in the transition to university life. First-year students who belong to dysfunctional families experience a higher mental burden and experience more symptoms of depression and anxiety (Wang et al., 2020; Caravaca-Sánchez et al., 2021).

On the other hand, we demonstrate that poor social support is associated with symptoms of anxiety and depression, and this association is independent of gender, age, and socioeconomic status. This result is consistent with other studies conducted in the university population (Alsubaie et al., 2019; Mackin et al., 2017). Social support is a network that provides material and psychological resources that buffer the pathogenic effects of stress (Xu and Wei, 2013). Lack of social support is associated with lower emotion, positive experiences, and lower perceived well-being (Aktekin et al., 2001; Viseu et al., 2018). Shao et al. (2020) found that medical students who had poor relationships with friends or classmates have higher levels of anxiety and depression; similarly, anxiety and depression correlated with social support. Students who have a social support network manifest lower perceived stress and a better ability to adapt positively to adverse situations (Islam et al., 2020; Shao et al., 2020). Likewise, it has been reported that social support mediates the relationship between anxiety and depression with the risk of suicide in university students, acting as a protective factor (Sun et al., 2020; Chu et al., 2021). The support of friends, families, colleagues, etc., would give students greater possibilities to deal with stressful and painful situations (Liébana-Presa et al., 2018).

## 5 Limitations and advantages

The major limitation of the study is its design. A cross-sectional study does not confirm the association between anxiety and depression with family dysfunction and support networks. Likewise, this study design is liable to information bias, mainly because some variables depending on the subjects’ perceptions. In addition, we do not measure other characteristics related to anxiety and depression in university students, such as personality traits, substance use, academic load, and recreational or relaxation activities, among others like interpersonal and environmental stressors. Among the strengths of this research, we have that this study is one of the few investigations analyzing anxiety and depression in a young population beginning university life. Also, it demonstrates the importance of family functionality and social support in this population.

## 6 Conclusion

Anxiety and depression are associated with moderate family dysfunctionality and the absence of social support. In addition, female sex, bad/deplorable economic situation, and marital status increase depression and anxiety in first-year university students. In this context, it is essential to implement university policies focused on promoting mental health in students and the accompaniment and permanent availability health team (physician, psychologist, social worker) that detects early signs and symptoms of these pathologies to make timely and effective interventions. These policies should include strategies to strengthen family functionality and social support, especially in students with social vulnerabilities.

## Data Availability

The raw data supporting the conclusions of this article will be made available by the authors, without undue reservation.
